# Genetic-Phenotype Analysis of *Bifidobacterium bifidum* and Its Glycoside Hydrolase Gene Distribution at Different Age Groups

**DOI:** 10.3390/foods12050922

**Published:** 2023-02-22

**Authors:** Xiaojing Wei, Leilei Yu, Chuan Zhang, Yongqing Ni, Hao Zhang, Qixiao Zhai, Fengwei Tian

**Affiliations:** 1 State Key Laboratory of Food Science and Technology, Jiangnan University, Wuxi 214122, China; 2 School of Food Science and Technology, Jiangnan University, Wuxi 214122, China; 3 National Engineering Research Center for Functional Food, Jiangnan University, Wuxi 214122, China; 4 School of Food Science and Technology, Shihezi University, Shihezi 832000, China

**Keywords:** glycoside hydrolase (GH), *Bifidobacterium bifidum*, 6’-sialyllactose, comparative genomics, genotype, phenotype

## Abstract

Human gut microbiota interfere with host development and aging. *Bifidobacterium* is a microbial genus found in the human digestive tract that has probiotic activities such as improving constipation and enhancing immunity. The species and numbers present change with age, but there has been limited research on probiotic gut microbiota at specific ages. This study analyzed the distribution of 610 bifidobacteria in subjects in several age groups (0−17, 18−65, and 66−108 y) using 486 fecal samples and determined the distribution of glycoside hydrolases based on genetic analysis of strains representing 85% of the *Bifidobacterium* species abundance in each age group. 6’-Sialyllactose is a major component of acidic breast milk oligosaccharides, which can promote human neurogenesis and bifidobacteria growth. Using genotypic and phenotypic association analysis, we investigated the utilization of 6’-sialyllactose by six *B. bifidum* strains isolated from subjects 0–17 and 18–65 y. A comparative genomic analysis of the six *B. bifidum* strains revealed differences in genomic features across age groups. Finally, the safety of these strains was evaluated by antibiotic gene and drug resistance phenotype analysis. Our results reveal that the distribution of glycoside hydrolase genes in *B. bifidum* varies with age, thus affecting the phenotypic results. This provides important insights for the design and application of probiotic products for different ages.

## 1. Introduction

Billions of bacteria colonize the human gut, with effects that are strongly linked to human health [1]. Different microbial communities colonize different parts of the human body [2], and the most densely colonized body part is the intestinal tract [3]. As humans age, the gut microbiota proceed through a series of phase changes. In early childhood, there is a dynamic gut microbial composition, while adults have a relatively stable flora, and the numbers of the intestinal microbiota in the elderly gradually decrease and health-promoting functions decline [4,5]. There is a close association between age-related changes in immunity, dysbiosis, disease, and probiotic-based products, and utilizing this association in combating age-related disease is an attractive proposition [6]. Some studies have proposed modifying the gut microbiome with live microbes, and clinical trials have demonstrated age-dependent beneficial effects of the consumption of *Lc*Z [7,8]. Given the importance and complexity of the gut microbiota, it is critical to gain an understanding of compositions, patterns, and the laws of intestinal microecology to better understand human health and disease.

Intestinal physiological changes due to age-related factors have an inevitable impact on the structure of the gut microbiota. Bifidobacteria are dominant microorganisms colonizing the gut in early life, with a strong competitive advantage due to an ability to break down human milk oligosaccharides (HMOs) [9,10]. The predominant *Bifidobacterium* species in the early infant gut is *Bifidobacterium breve*, along with *B. bifidum* and *B. longum* subsp. *infantis* [11,12]. Macrogenomic sequencing revealed a high abundance of *B. longum* subsp. *longum* and *B. pseudobulbarum* in the gut of breastfed infants [13,14]. With a decrease in breastfeeding and a gradual increase in solid food intake, the composition of the infant intestinal microbiota begins to shift toward that of the adult gut [15]. The composition of *Bifidobacterium* species in the adult gut is more complex. Among the *Bifidobacterium* species present are *B. longum* subsp. *longum*, *B. adolescentis*, and *B. catenulatum*, as well as a lower-abundance *B. bifidum* and *B. breve* [16]. The numbers of bifidobacteria in the intestinal microbiota of the elderly are generally lower than in that of non-elderly adults, and the diversity of bifidobacteria is significantly reduced primarily due to a loss of *B. adolescentis*, *B. longum* subsp. *longum*, and *B. angulatum* [17].

*Bifidobacterium* uses non-digestive carbon sources and has cross-intertrophic effects on nutrients with other intestinal microbiota to maintain gut homeostasis. More than 13% of the genes in the bifidobacteria genome are involved in carbohydrate metabolism, with glycoside hydrolase being the most important component. Given the various glycoside hydrolases carried by different species, the types of carbohydrates in the gut have a significant impact on *Bifidobacterium* composition [18]. Through the analysis of bacterial comparative genomics, the relationship between their genomic and phenotypic features can be determined, thus facilitating further examination of the related molecular mechanisms. The whole genome sequencing and bioinformatics analysis of *B. bifidum* PRL2010 published in PNAS in 2010 revealed the unique degradation and utilization mechanism of *B. bifidum* mucin oligosaccharides, helping people understand the unique mechanism of *B. bifidum* intestinal adaptation [19,20]. Currently, the available bifidobacteria genome in the NCBI database allows us to conduct genomic analyses of strain evolutionary relationships, functional conservation, and variability. Arboleya et al. [21] reported genomic data for *B. longum*, revealing a rich complement of glycosyl hydrolase genes leading to strong polysaccharide utilization ability. Using comparative genomics, Duranti et al. [22] demonstrated a lack of genes for metabolism of host-derived polysaccharides (such as HMOs or mucin) in the *B. adolescentis* genome, explaining at a genetic level why *B. adolescentis* is abundant in the adult intestine. Using comparative genomic analysis, Lu et al. [23] found that core enzymes isolated from *B. bifidum* strains from different ecological niches accounted at a molecular level for an ability to metabolize host-derived polysaccharides.

In contrast to other *Bifidobacterium* species, *B. bifidum* has extracellular glycosidases that can degrade many host-derived glycans, including HMOs, glycan chains of high-molecular-weight glycoproteins, and glycosphingolipids [24,25]. Sialic acids are constituents of HMOs and include sialyloligosaccharides [26] such as 6′-sialyllactose (6′-SL) and 3′-SL as well as N-acetylneuraminic acid (Neu5Ac). These compounds play an important role in nerve development. The extracellular degradation of sialylated HMOs by *B. bifidum* JCM1254 is one example of a broader metabolic activity of bifidobacteria [27]. *B. bifidum* not only utilizes polysaccharides in the gastrointestinal tract through its own GH activity but also benefits the growth of other microorganisms by breaking down polysaccharides into monosaccharides. Comparative studies of the genomes of *B. bifidum* strains require additional strains for validation. Existing studies on strain–age relationships are also limited. Various studies have shown that an imbalance in human intestinal bifidobacteria can have adverse effects on health. As the ability of bifidobacteria to metabolize plant- and host-derived carbon sources is the basis of their long-term colonization of the intestinal tract, it is critical to investigate the carbohydrate utilization abilities of bifidobacteria present at different stages of human life. The data obtained can be used as a reference for targeted intervention to assist the intestinal growth of *Bifidobacterium* and to provide new ideas for designing probiotic products for different age groups. There is a need to collect more genome-level information on *Bifidobacterium* and conduct in-depth comparative analyses to better understand the relationship between the intestinal microbiota, host age, and health.

## 2. Materials and Methods

### 2.1. Bacterial Screening

The distribution of 610 bifidobacteria from three age groups in the strain library was counted (Table 1). Reference strains were from the food biotechnology research center of Jiangnan University. The collection of fecal samples was approved by the Ethics Committee of Jiangnan University, China (SYXK 2012–0002). Based on the occurrence of *Bifidobacterium* spp. in each age group, we selected strains representing a summed abundance of bifidobacteria in each age group of >85%, comprising five species each for the age 0–17 y and 18–65 y groups, and four species for the age 66–108 y group. Three bifidobacterial strains were selected at random for each species, comprising a total of 42 strains. Strains preserved at −80 °C were thawed and inoculated onto 2% mMRS culture medium and grown in an anaerobic incubator for 24–48 h. Purified single colonies were taken up in 0.1 mL medium, incubated for 48 h, then grown in mMRS liquid medium supplemented with 0.5% L-cysteine for 24–48 h. Samples (1 mL) were centrifuged (6000 rpm for 3 min). The supernatants were discarded, and pellets were washed twice with 1.5 mL sterile water. Pellets were resuspended in 1 mL sterile water, and samples were used as templates for strain identification. 16S rRNA PCR amplification was performed as previous reported [28]. Amplified products were subjected to nucleic acid electrophoresis, and the single band on the agarose gel was excised and sent to GENEWIZ Co., Ltd. (South Plainfield, NJ, USA). for sequencing. 16S rDNA sequences were used for NCBI BLAST alignment. 

### 2.2. Genomic DNA Extraction

Strains were cultured in mMRS liquid medium at 37 °C for 24–48 h and then pelleted for genomic DNA extraction. Genomic DNA was extracted using a bacterial DNA extraction kit (Omega Bio-Tek, Norcross, GA, USA) following the manufacturer’s instructions. The extracted genomic DNA was tested for quality using agarose gel electrophoresis (1% gel concentration), purity using a UV spectrophotometer, and concentration using a QubitTM 4 fluorometer and a Qubit DNA Assay Kit (Life Technologies, Carlsbad, CA, USA).

### 2.3. Genome Sequencing, Assembly, and Annotation

The genomes of 42 *Bifidobacterium* strains were sequenced at Majorbio BioTech Co., Ltd. using the Hiseq X Ten platform (Illumina, San Diego, CA, USA). Qualified bacterial genomic DNA (700 ng) was used for the construction of sequencing libraries using an NEB Next Ultra DNA Library Prep Kit (New England Biolabs, Ipswich, MA, USA). After library construction, library sequences were clustered using the HiSeq 4000 PE Cluster Kit (Illumina) and sequenced using the Hiseq 4000 platform (Illumina). A paired-end read of 150 bp was selected for the sequencing library. High-quality paired-end reads were spliced using SOAPdenovo2, and internal gaps were filled using GapCloser [29]. The open reading frame (ORF) of Glimmer 3.02 was used, and gene sequences were translated into amino acid sequences using the Transeq tool in EMBOSS6. Following the method of Pan et al. [30], functional gene annotation of predicted genes (COG and NR databases) was performed using Prokka or BLASTX. Pan-genome and core gene analyses were performed using PGAP-1.2.1. Glimmer 3.02 and GeneMarkS [31] were used to obtain the GC content, the raw amino acid sequence (.faa), and the nucleotide sequence (.fna) of each genome. Results were base-annotated using the Kyoto Encyclopedia of Genes and Genomes (KEGG), Clusters of Orthologous Genes (COG), and Swiss-Prot databases. Protein sequences for all strains were obtained using the dbcan2 metagenome tool (http://bcb.unl.edu/dbCAN2/, accessed on 18 March 2021). Carbohydrate-active enzyme annotations were performed using Hmmer. Annotated glycoside hydrolases were coded and classified using EC data (http://www.ebi.ac.uk/, accessed on 18 March 2021).

### 2.4. Determination of the Ability of Bifidobacteria to Utilize 6’-Sialyllactose

Frozen bifidobacterial cultures (containing carbohydrate-active enzymes in the GH33 family) were added to 5 mL mMRS medium (2% (*v*/*v*) inoculation), placed in an anaerobic workstation, and cultured at 37 °C for 24–36 h. After 3 generations of continuous subculturing, bacteria were collected via centrifugation, washed, and resuspended in an equal volume of sterilized normal saline. The bacterial suspension was inoculated into culture medium with unique carbon sources and the resuspended cultures were added to three 96-well plates (2 mL capacity) and incubated in a fully automated enzyme labeling instrument in an anaerobic workstation at 37 °C for 40 h. OD_600_ values were measured at every other period. The experiment was conducted in triplicate. 6′-Sialyllactose used in the carbon source medium was purchased from Shanghai HuicH Biotechnology Co., Ltd. (Shanghai, China) and had a purity of 98% by HPLC and a degree of polymerization of 23, 6′-Sialyllactose was added to the medium at a final concentration of 0.5% (*w*/*v*) [21]. 

### 2.5. Comparative Analysis of the Genomes of Six B. bifidum Strains from Subjects of Different Ages

Orthologs from six *B. bifidum* strains were defined using OrthoMCL with default parameters [32]. The core gene sequences chosen were aligned using PGAP-1.2.1. Pairwise average nucleotide identity (ANI) values of *B. bifidum* genomes were calculated and visualized using R (Version 3.6.1). Orthologous proteins, carbohydrate-active enzymes, virulence factors, and antibiotic factors were annotated using the COG, CAZyme, Gene Ontology (GO), ANI Calculation, comprehensive antibiotic research (CARD), and Virulence Factor (VFDB) databases using BLAST with default parameters. Information about the six *B. bifidum* strains and quality data for each genome (genome size, GC content, genomic level, and gene number) [33].

### 2.6. Antibiotic Resistance Gene and Phenotype Analysis of B. bifidum

Antibiotic resistance genes were predicted to be present in the *B. bifidum* genome by comparing the amino acid sequences of six *B. bifidum* strains to those registered at CARD (http://arpcard.mcmaster.ca, accessed on 25 April 2021) [34]. The antibiotic susceptibility of *B. bifidum* strains was determined using a disk diffusion method, as recommended by the Clinical and Laboratory Standards Institute (CLSI) (CLSI, 2012). Drugs and concentrations were taken from the experimental method by Jin et al. [35], and all drugs were purchased from Sangon Biotech Co., Ltd. (Shanghai, China). *B. bifidum* strains were categorized as resistant, susceptible, or intermediate, according to inhibition zone diameters.

### 2.7. Statistical Analysis

Growth curve data were analyzed and visualized using GraphPad Prism 9.1. Excel 2021 was used to analyze the distribution of bifidobacteria. R (Version 3.6.1) software was used to compare genomic data. The heat map of *Bifidobacterium* glycoside hydrolase gene distributions was constructed using Helm V1.0.s [36].

## 3. Results

### 3.1. Bifidobacteria Distribution at Various Ages

**Figure 1:**
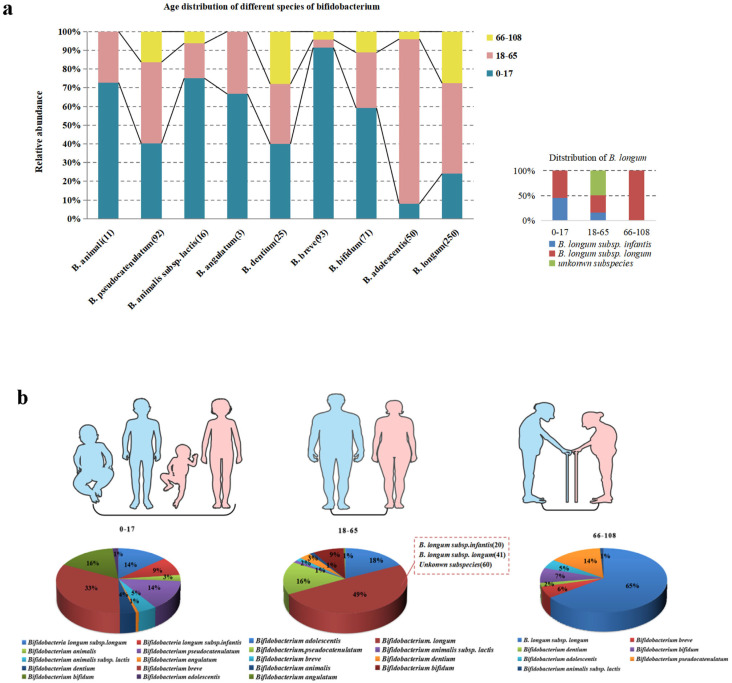
The distribution of the bifidobacteria isolated. (**a**) Distribution of bifidobacteria by age groups. (**b**) Proportions of different kinds of bifidobacteria in different age groups.

The identification of gut microbiota using 16S rRNA sequencing is only accurate at a genus level, while *GroEL* sequencing can distinguish bifidobacteria at a species or subspecies level. In this study, 610 strains of bifidobacteria were identified in 446 fecal samples using *GroEL* sequencing. The numbers of bacterial strains in subjects from age groups 0–17, 18–65, and 66–108 y was 260, 244, and 106, respectively. A total of nine species of *Bifidobacterium* was identified across all samples (Figure 1a), of which 250 *B. longum* strains included two clearly identified subspecies: *B. longum* subsp. *infantis* and *B. longum* subsp. *longum*; while unidentified subspecies of *B. animali* and *B. augulatum* were not isolated from the 66–108 age group but were present in the other two age groups. *B. breve* was the most abundant bifidobacterium in the 0–17 y age group, accounting for 33% of all *Bifidobacterium* species in this group. Other species with high abundance were *B. bifidum*, *B. longum* subsp. *longum*, *B. longum* subsp. *infantis*, and *B. pseudocatenulatum*. The most abundant species in the 18–65 y age group was *B. longum*, accounting for 49% of the bifidobacteria in this group (including two well-identified subspecies, *B. longum* subsp. *infantis* and *B. longum* subsp. *longum,* as well as an unknown subspecies), followed by *B. adolescentis* (18%), *B. pseudocatenulatum*, and *B. bifidum*. The most abundant species in the 66–108 y age group was *B. longum* subsp. *longum*, accounting for 65% of the bifidobacteria isolated from subjects in this age group, which was followed by *B. pseudocatenulatum* (14%), *B. bifidum,* and *B. breve* (Figure 1b).

### 3.2. Glycoside Hydrolase Genes of Bifidobacterium Species Was Studied at Various Ages

**Figure 2:**
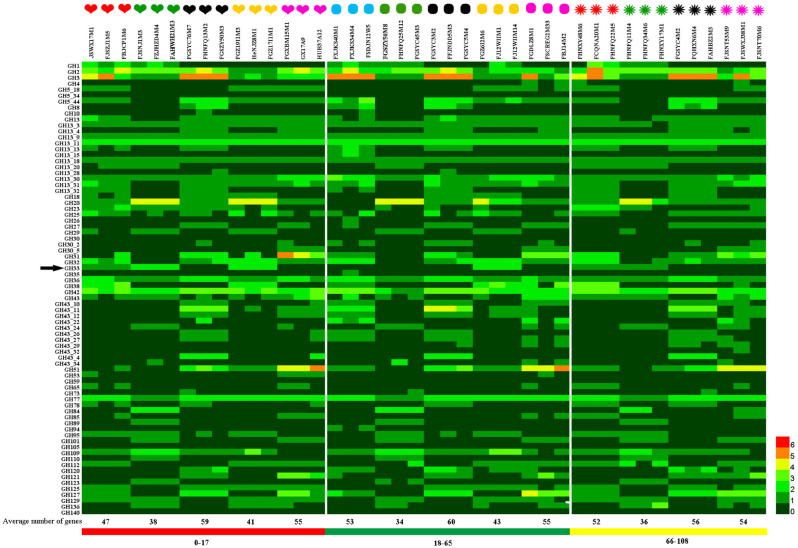
Analysis of the glycoside hydrolase genes from *Bifidobacterium* spp. isolated from subjects in different age groups. The heatmap shows the number of ORFs annotated as GH for each GH family (*y*-axis) and each genome (*x*-axis). The arrow marks GH33.

An ability to utilize carbon sources in the gut is an important factor in determining the abundance of gut microbes. The hydrolysis of carbohydrates in the gut by GHs is a key step allowing utilization by microbes, whereby the substrate specificities and numbers of glycoside hydrolases encoded by microorganisms determine the range of available carbon sources. To evaluate the carbohydrate utilization ability of bifidobacteria from subjects in different age groups, we selected strains representing a summed abundance of bifidobacterial species in each age group of >85%, including five species from the 0–17 and 18–65 y age groups and four species from the 66–108 y age group. A total of 42 strains of *Bifidobacterium* were selected. We used the dbCAN server to assess the presence of genes encoding glycosyl hydrolases and annotated genes based on GH hidden Markov models (HMMs) generated from data from the CAZy database. Forty-nine families of glycoside hydrolase were found encoded in the genome. Heat maps are shown in Figure 2. The heart icon represents the 0–17 y age group, the oval icon represents the 18–65 y age group, and the firework icon represents the 66–108 y age group. Red represents *B. breve*, green represents *B. bifidum*, black represents *B. pseudocatenulatum*, yellow represents *B. longum* subsp. *infantis*, blue represents *B. adolescentis*, and purple represents *B. longum* subsp. *longum*.

Statistical analysis showed that there was no significant difference in the average number of glycoside hydrolase genes among species isolated from different age groups. However, it is of interest to note that *B. pseudocatenulatum* has the highest average number of carbohydrase genes among species taken from all age groups, which is followed by *B. longum* subsp. *longum*. The average number of carbohydrase genes in *B. bifidum* was the lowest among species taken from all age groups, being 38, 34, and 36 in samples from subjects in the 0–17, 18–65, and 66–108 y age groups, respectively. The genomes of all strains encoded the GH2, GH13, GH36, GH42, and GH77 families of glycosidase hydrolases, and they were predicted to be primarily amylases as well as some β-galactosidases that are involved in degrading most of the non-digestible carbon sources in staple foods and dairy products. These glycosidases are essential for the proliferation of *Bifidobacterium* and can be considered core bifidobacterial glycoside hydrolases. *Bifidobacterium* genomes also encode glycoside hydrolases associated with the degradation of host-derived carbon sources, and these are encoded in the largest number in the genomes of *B. bifidum* and *B. longum* subsp. *infantis*. For example, *B. bifidum* and *B. longum* subsp. *infantis* encode an average of four β-hexosaminidase genes in the GH20 family, but *B. adolescens* does not possess genes for this enzyme. α-Fucosidases of the GH95 family are encoded in the genomes of *B. bifidum*, *B. longum* subsp. *infantis*, *B. breve*, and some strains of *B. pseudocatenulatum*, and they are involved in the degradation of 2′-fucosyllactose. *B. longum* subsp. *infantis* encodes an average of two sialidases from the GH33 family, which is possibly related to the degradation of sialyllactose or other sialic-acid-modified glycans in HMOs. The three strains of *B. bifidum* encode an average of two sialidases from the GH33 family in ages 0–17 y, but *B. bifidum* from subjects of age 18–65 y does not encode these enzymes, and there is only an average of one sialidase from the GH33 family in samples from the 66–108 y age group. Different glycoside hydrolases endow *Bifidobacterium* with specific carbon source utilization abilities, provide a unique ecological adaptation mechanism, and affect interactions with other species.

### 3.3. Determination of the Ability of Bifidobacteria to Utilize 6’-Sialyllactose

**Figure 3:**
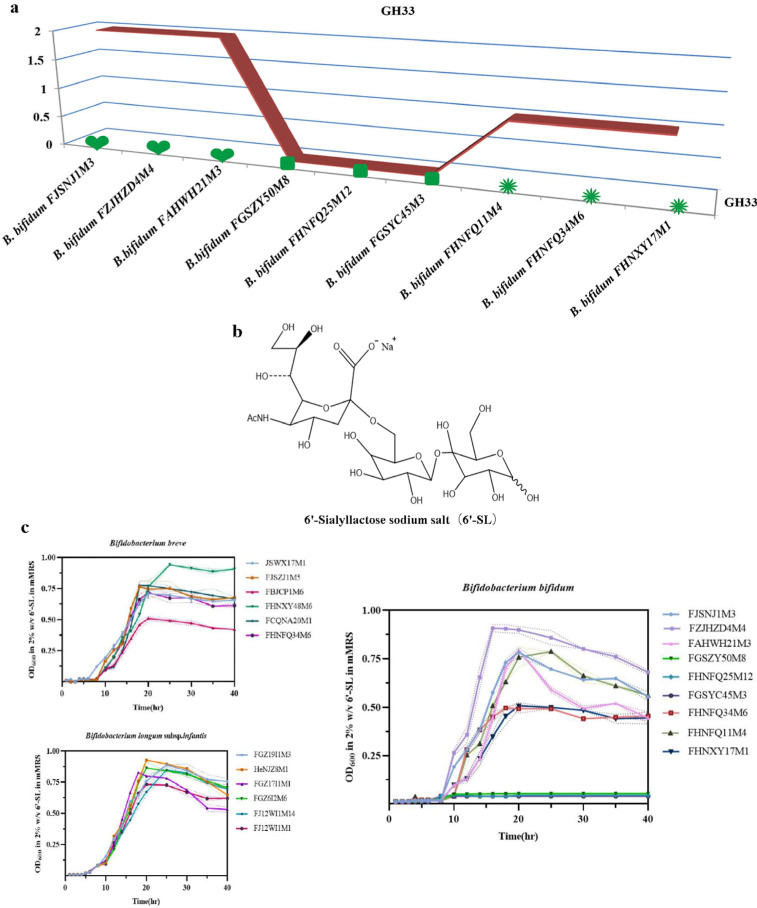
6’-Sialyllactose as a key carbon source for *Bifidobacterium* growth. (**a**) Numbers of GH33 family glycoside hydrolase enzymes in *B. bifidum* isolated from different age groups; (**b**) Chemical structure of HMO 6’-SL generated; (**c**) Growth in 6′-SL-containing medium for *Bifidobacterium* strains containing the GH33 family gene encoding glycoside hydrolase.

6’-Sialyllactose is primarily found in human milk and mammalian tissues. It has many biological functions and is an important component of glycoproteins and glycolipids with functions in cell recognition and immune responses. In this study, 6’-sialyllactose (structure in Figure 3b) was used as a sole carbohydrate source to evaluate the utilization phenotype for 6’-SL utilization of different *Bifidobacterium* species containing the GH33 gene (Figure 2). The results of the phenotypic experiments are given in Table 2. All six strains of *B. breve* were able to utilize 6’-SL, although strain FBJCP1M6 was less able to use it than the other strains. The six strains of *B. longum* subsp. *infantis* were able to grow well in the medium with 6’-SL as a sole carbon source. The phenotypic utilization of 6’-SL by *B. bifidum* in different age groups was mainly studied. Six *B. bifidum* strains from the 0–17 and 65–108 y age groups were able to use this acidic oligosaccharide, but the three *B. bifidum* strains from subjects in the 18–65 age group showed no obvious growth consistent with the genotype of *B. bifidum* in terms of glycoside hydrolases from the GH33 family, as shown in Figure 3a. The corresponding growth curves of these strains are shown in Figure 3c. Therefore, further investigation was conducted to determine whether three *B. bifidum* strains, FJSNJ1M3, FZJHZD4M4, and FAHWH21M3, from subjects in the 0–17 y age group (encoding an average of two sialidases from the GH33 family) and three *B. bifidum* strains from subjects in the 18–65 age group that lacked genes for the GH33 family of sialidases, were different at a genomic level.

### 3.4. Comparison of Genomes between Six Strains of B. bifidum from Subjects of Different Ages

**Figure 4:**
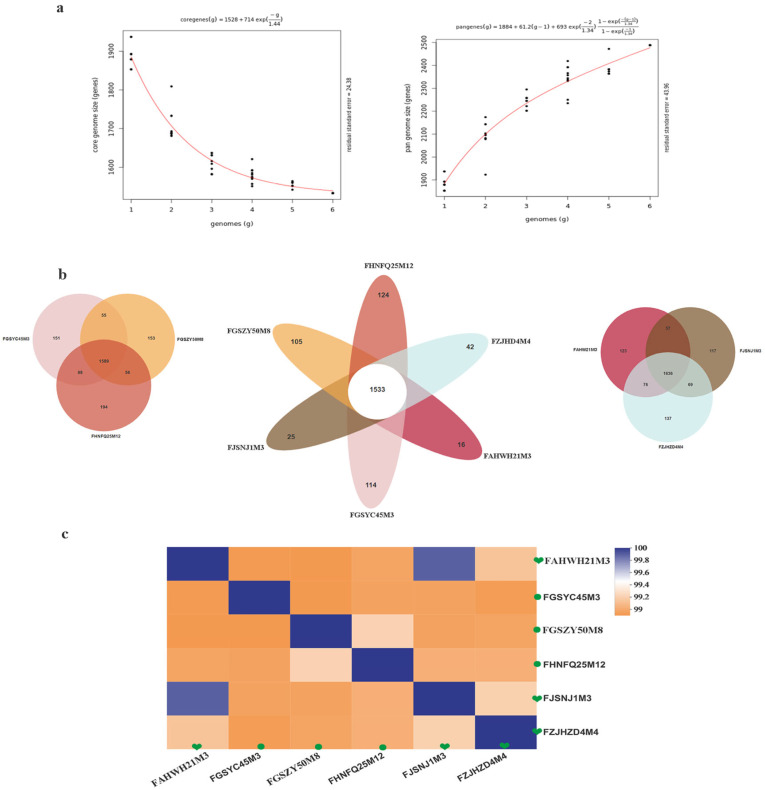
Comparative genomic and phylogenetic analyses of six *B. bifidum* strains. (**a**) Pan-genome and core genome; (**b**) Venn diagrams based on the homologous genes of six *B. bifidum* strains isolated from human feces from different age groups. (**c**) ANI value among six *B. bifidum* strains.

Comparative genomics was used to explore differences in the genomes of six strains of *B. bifidum* strains from subjects in the 0–17 and 18–65 y age groups. As shown in Table 3, the average genome size for the six bifidobacteria is 2.14 MB, and the average GC content is 62.6%. GC content and genome size are considered to be closely related to bacterial genome evolution and energy metabolism. Changes in the bacterial genome size may explain changes in bacterial carbohydrate metabolism and amino acid metabolism. The genomes of six *B. bifidum* strains were analyzed for pan- and core genes (Figure 4a). The results showed that the number of pan-genes increased with the number of strains, while the number of core genes tended to stabilize. When the sixth strain was added, the number of pan-genes stabilized at 2488 and the number of core genes reached 1533. The asymptotic trend of the pan-genomic curve may indicate that *B. bifidum* has an open pan-genome. The specific core genes and homologous core genes of *B. bifidum* strains were determined, and a Wayne diagram was drawn (Figure 4b). The analysis using OrthoMCL showed that the six strains had 1533 common core genes with numbers of unique specific genes, ranging from 16 to 124. Although there are some differences in age-specific genes, the results showed that the specific genes in the three strains of *B. bifidum* strains from subjects in the 0–17 y age group were present in significantly lower numbers than those in *B. bifidum* from subjects in the 18–65 age group.

**Figure 5:**
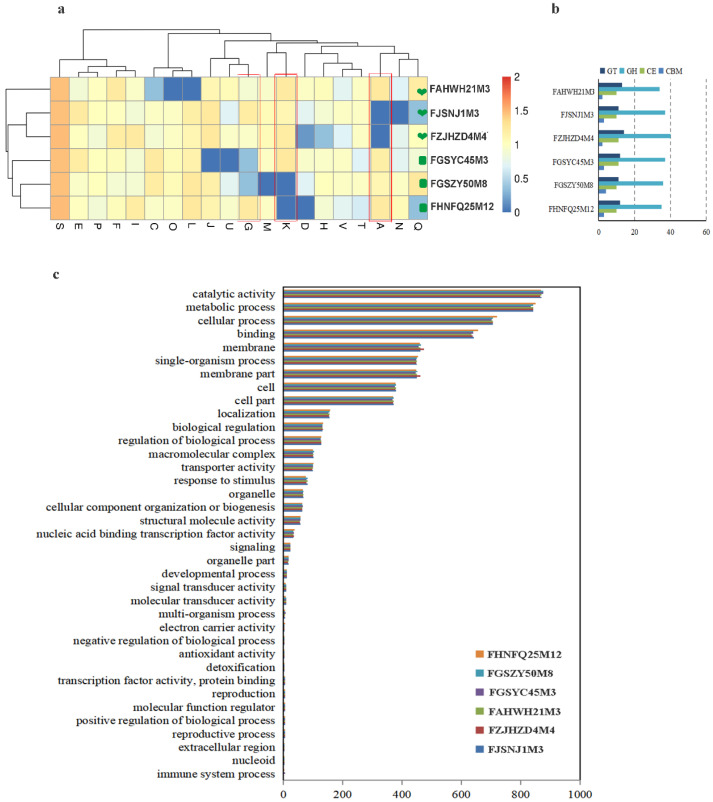
Functional gene analysis of six strains of *B. bifidum*. (**a**) Functional assignment of the core genome based on the COG database; (**b**) The number of genes in four families of carbohydrate-active enzymes from different strains of *B. bifidum*; (**c**) Functional assignment of the core genome based on the GO database.

Between-strain ANI values can determine the similarity of two genetic sequences at a genomic level. ANI values for the six *B. bifidum* strains were calculated, and it was found that the distribution of ANI values within the *B. bifidum* group was consistent, ranging from 99% to 100%. As shown in Figure 4c, ANI values were more similar within a single age group. The results show that *B. bifidum* genomes from subjects in the 0–17 y age group are more similar to each other than to those of *B. bifidum* strains in the 18–65 age group. To distinguish whether the distribution of functional genes in the *B. bifidum* genome differed among samples from the different age groups, the six *B. bifidum* genomes were functionally annotated using the COG database and analyzed statistically for the two age groups (Figure 5a). By comparing the distribution of COG genes in the two age groups, it was found that gene classes J (Translation, ribosomal structure), G (Carbohydrate transport), M (Cell wall biogenesis), K (Transcription), and T (Signal transduction) were higher in the genome of *B. bifidum* from subjects of age 0−17 y than from subjects of age 18−65 y, while gene classes A (RNA processing), N (Cell motility), U (Intracellular trafficking), O (protein turnover), and L were lower in subjects of age 0–17 y than in those of ages 18−65 y. Carbohydrate metabolic pathway expression was significantly greater in *B. bifidum* from subjects aged 0–17 y than in *B. bifidum* from subjects aged 18–65 y. *B. bifidum* in the two age groups showed no significant differences in GO enrichment. Most of the differentially distributed genes had catalytic activity and metabolism-related functions (Figure 5c). Six *B. bifidum* strains encoding a large number of carbohydrate-active enzymes (CAZymes), including GTs, GHs, CEs, and CBMs, of which, GHs were the most abundantly distributed (Figure 5b).

### 3.5. Antibiotic Resistance Gene and Phenotype Analysis in B. bifidum

**Figure 6:**
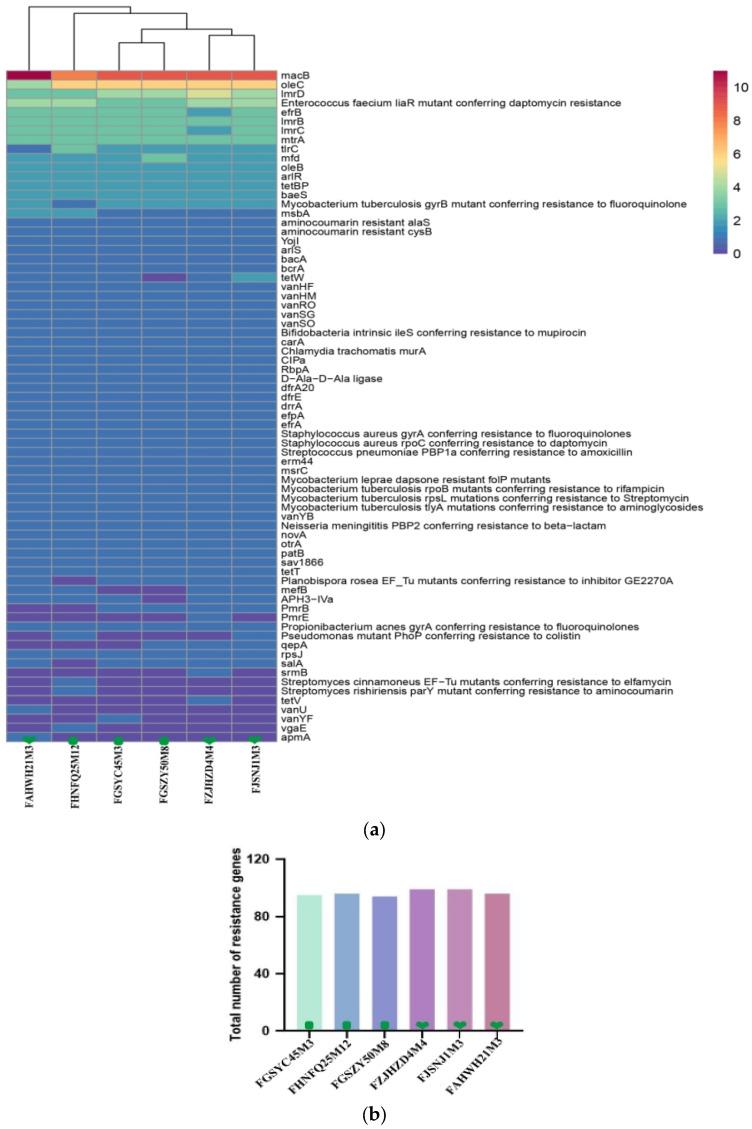
Genotype−phenotype analysis of the antibiotic resistance of *B. bifidum*. (**a**) Clustering heat map analysis of antibiotic resistance genes in *B. bifidum*; (**b**) The total number of resistance genes in six *B. bifidum* strains.

Antibiotic tolerance is important in maintaining the abundance of gut symbiotic bacteria, but the horizontal transfer of resistance genes between gut microbes may lead to the generation of deleterious antibiotic-resistant pathogenic bacteria. Using antibiotic resistance testing, this study evaluated the safety of six strains of *B. bifidum* obtained from subjects of different ages, providing a reference for future application in the probiotics industry. The *B. bifidum* genomes were annotated using the CRAD database to determine whether they contained potential antibiotic resistance genes. As shown in Figure 6a, each strain carried an average of 97 antibiotic resistance genes of 73 different types, including a macrolide resistance gene (macB), which had the highest content in all strains, which was followed by the lincosamide resistance genes (lmrB and lmrD), aminoglycoside resistance genes (baeS), and glycopeptide resistance genes (vanHF and vanHM). By comparing the total numbers of resistance genes in six *B. bifidum* isolates from subjects of different ages, the result showed that the resistance genes in *B. bifidum* isolates from the 0–17 y group were higher than those from the 18–65 age group (Figure 6b). Among these, the number of resistance genes in *B. bifidum* FZJHZD4M4 and *B. bifidum* FJSNJ1M3 was the highest (99), and that in *B. bifidum* FGSZY50M8 was the lowest (94). Table 4 shows the antibiotic susceptibility of the six *B. bifidum* strains to 18 antibiotics. In this study, the tested strains were resistant to ciprofloxacin, trimethoprim, neomycin, kanamycin, streptomycin, aztreonam, and erythromycin but were fully or moderately susceptible to penicillin, ampicillin sodium, amoxicillin, chloramphenicol, tetracycline, rifampicin, ceftizoxime, teicoplanin, vancomycin, and oxacillin. These was semi-tolerance to other antibiotics. The results indicated that *B. bifidum* strains had different responses to the antibiotics; however, most of them were susceptible to ten different antibiotics. The antibiotic resistance phenotypes were highly consistent with their genotypes.

## 4. Discussion

*Bifidobacterium* species are among the earliest microorganisms to colonize the human intestine and play important roles in maintaining host health.

*Bifidobacterium* species can affect various disease states, for example, by exercising anti-tumor effects. In a recent study, oral administration of *Bifidobacterium* to mice achieved an anti-cancer effect of similar magnitude to that of anti-PD-L1 therapy, while combined treatment almost completely inhibited tumor growth [37]. *Bifidobacterium* and derived preparations have been reported to regulate inflammatory bowel disease by altering the diversity of the intestinal microbiota, regulating the intestinal immune response, and secreting anti-pathogenic substances [38]. The establishment of *Bifidobacterium* in the intestine can lead to an increase in lactic acid production and reduce the pH of the intestine, inhibiting the propagation of harmful bacteria such as *Escherichia coli* and *Clostridium* spp. They can also optimize the physical and chemical environment of the intestine and inhibit the initiation and development of colon cancer induced by azomethane oxide [39]. *Bifidobacterium* spp. are also capable of reducing blood lipid levels, regulating the intestinal environment, exercising anti-aging effects, and assisting defecation [40]. *Bifidobacterium* is mainly passed via mother-to-child vertical transmission at birth [41]. Common bifidobacteria in the intestinal tracts of infants include *B. bifidum*, *B. longum* subsp. *infants*, and *B. breve* [42], while the common *Bifidobacterium* species in the adult gut are *B. adolescentis*, *B. pseudobulbarum*, and *B. longum* subsp. *longum* [43]. Common bifidobacteria species in the intestine of the elderly are *B. longum* subsp. *longum*, *B. pseudobulbarum* and *B. bifidum*. Aging, which may be accompanied by a change in carbon source intake, is one of many factors that can influence the species composition of *Bifidobacterium* in the human intestinal tract [44]. According to the World Health Organization, human life can be divided into five stages: 0 to 17 years, 18 to 65 years, 66 to 79 years, 80 to 99 years and over 100 years. In this work, 610 *Bifidobacterium* strains were divided into three groups based on subject age. The results showed that there were 260 *Bifidobacterium* strains from subjects in the 0−17 y age group, with *B. breve*, *B. bifidum*, *B. longum* subsp. *longum*, *B. longum* subsp. *infantis*, and *B. pseudobulbarum* having the highest abundance. From subjects of age 18–65 y, we isolated 244 *Bifidobacterium* strains, with *B. adolescentis*, *B. pseudobulbarum*, *B. longum* subsp. *longum*, and *B. longum* subsp. *infantis* having the highest abundance. A total of 106 strains were found in fecal samples from subjects in the 66−108 y age group, of which the abundance of *B. longum* subsp. *longum* accounted for 65%, which was followed by *B. pseudobulbarum* and *B. bifidum*. These findings are consistent with previous results [45].

More than 13% of the homologous gene family clusters in the bifidobacterial genome are associated with carbohydrate metabolism [20]. This glycemic genotype allows *Bifidobacterium* to metabolize various carbohydrates that cannot be digested by host enzymes [46], providing a competitive advantage for *Bifidobacterium* in colonizing the complex intestinal environment. Glycoside hydrolases are enzymes that catalyze carbohydrate hydrolysis. In recent years, the number of GHs represented in the CAZy database has increased almost exponentially [47]. When He et al. [48] studied the microbial samples in the rumen of sheep using macrotranscriptional sequencing, it was found that more than half of the GHs were located in the CAZymes group. *B. bifidum* has many extracellular glycosidases that can degrade host-derived glycans, including human milk oligosaccharides and high-molecular-weight carbohydrate chains [24]. *Bifidobacterium* enzymes can be used in the food industry, especially in the production of glycosylated products. For example, *Bifidobacterium* can be added in the preparation of yogurt to directly synthesize oligomeric galactose using lactose as a substrate [49]. The present study focused on carbohydrate-active enzymes in *Bifidobacterium* from subjects in different age groups and the annotation and distribution of the abundance glycoside hydrolase genes. The results showed no obvious differences in the glycoside hydrolase gene distribution between samples from subjects of different ages, but there were significant differences in *Bifidobacterium* species distribution. Across all age groups, *B. pseudobulbarum* had the largest number of glycoside hydrolase genes, with an average of 58 genes, which was followed by *B. longum* subsp. *longum* (55) and *B. breve* (51). The *B. bifidum* genome coded for the smallest number of GH genes, with an average of 36. *B. bifidum* is one of the earliest bacteria to colonize the intestinal tracts of infants, and it has high abundance in the infant intestinal tract. Some specific mechanisms in *B. bifidum* may be responsible for the highly competitive nature of this taxon in the infant intestine, allowing it to persist in this special environment. Previous studies have found that the combined effects of N-acetylneuraminic lyase (nanA), N-acetylmannosamine kinase (nanK), and N-acetylmannosamine isomerase (nanE) affect the degradation of sialic acid in some *Bifidobacterium* species [50]. The presence of sialidase was confirmed in the genomes of strains including *B. longum* subsp. *infantis* ATCC15697, *B. breve* UCC2003, and *B. bifidum* PRL2010 [51]. Kiyohara et al. [27] first reported that the *B. bifidum* JCM1254 exosialidase SiaBb2, an extracellular enzyme located on the membrane and belonging to the GH33 family, can act on sialylated oligosaccharides and promote release of free sialic acid. Yu et al. [52] showed that *B. longum* subsp. *infantis* JCM7009 and JCM7011 can efficiently utilize 3′-SL and 6′-SL using neuraminidase and ultimately produce lactate and short-chain fatty acids. Other results showed that *B. bifidum* extracellular sialidase promotes the utilization of sialylated carbohydrates with cross-feeding of free sialic acid to other *Bifidobacterium* strains. *Bifidobacterium* can grow in media containing SL as the main carbon source as it has sialidase- and galactosidase-encoding genes that allow for the cleavage of the relevant glycosidic bonds [53]. To assess this, we selected 21 *Bifidobacterium* strains from subjects of different ages, including *B. breve* (6 strains), *B. longum* subsp. *infantis* (6), and *B. bifidum* (9) and assessed the gene distribution of the GH33 family of glycosidases. All the strains were subjected to an in vitro 6′-SL utilization test. The results showed that all the *B. longum* subsp. *infantis* and *B. breve* samples grew well with 6′-SL as sole carbon source, and most of the *B. bifidum* strains (other than those isolated from subjects in the 18–65 age group) could also utilize 6′-SL (Table 2), which is consistent with previous reports [52]. By correlating the presence or absence of genes and the growth or nongrowth patterns of 21 *Bifidobacterium* strains with 6′-SL as sole carbon source, we found that the phenotypic experiment results were consistent with the genotypic predictions. The distribution of genes in the GH33 family of sialidases varied for *B. bifidum* across samples from different age groups, with no GH33 genes detected in strains isolated from the 18–65 y age group, and only a few genes detected in strains from the 0–17 y (2) and 66–108 y (1) age groups. The development of genomic tools provides strong support for understanding the diversity and functional characteristics of bacterial strains. We performed comparative genomic analyses on six strains of *B. bifidum* from subjects in the 0–18 y and 18–65 y age groups and determined the carbohydrate metabolic capacity. The average genome size for the six *B. bifidum* strains was 2.17 Mb, which is consistent with previous reports [54]. The pan-genome and core genome results suggested that the six *B. bifidum* strains have an open pan-genome. Average nucleotide identity is a standard method to determine whether a particular strain belongs to a reference species or whether a subspecies exists, whereby a threshold of 96% is used as a species boundary [55,56]. The heatmap of ANI values showed that the average nucleotide identity was higher in strains obtained from the same age group (Figure 4c). Furthermore, by annotating the core genes, the functions of transcription, defense mechanisms, and general function prediction of *B. bifidum* were revealed. We found differences between the six *B. bifidum* strains obtained from subjects in the 0−17 and 18−65 y age groups in terms of core and pan-genomes, ANI, carbohydrate utilization enzymes, COG, and antibiotic resistance. GO analysis, antibiotic resistance genes, and antibiotic resistance phenotypes did not show differences with age. Even so, age-related factors may be important in developing appropriate probiotics. 

In contrast to the other *Bifidobacterium* species, *B. bifidum* has been shown to utilize host-derived carbohydrates, especially human milk oligosaccharides [57] and mucin [58]. In vitro and animal studies support the proposal that various extracellular proteins produced by *B. bifidum* are crucial for the interaction between strain and host. In recent years, numerous studies have confirmed the influence of gut microbiota on healthy aging and shown that the ability of intestinal microbiota to assist in fighting disease gradually decreases with age. This study found a loss of the GH33 family of glycosidase genes in strains isolated from the 18–65 y age group, possibly indicating a partial loss of metabolic capacity with age. Probiotics with particular functions may be useful as supplements for populations with specific needs. At present, the knowledge of the *B. bifidum* genome is limited, and more strains should be studied. Species numbers, subject ages, geographic regions, and other potentially relevant factors in existing studies remain limited. It is urgent to investigate the potential of age-specific probiotics at a genetic level. This study provides a reference for future application in probiotic development, taking into account factors including glycoside hydrolase types, phenotype–genotype relationships, and subject age.

## 5. Conclusions

In this study, whole genome sequencing was used to reveal the distribution of glycoside hydrolases in *Bifidobacterium*, with the results revealing differences in the distribution of the sialidase GH33 family in *B. bifidum* at different ages. Comparative genome and phenotypic studies were conducted, and the studies confirmed that the distribution of glycoenzyme genes in *B. bifidum* varied with age, affecting the phenotypic outcomes. It is critical to investigate the genomic and carbohydrate utilization properties of *Bifidobacterium* present in the digestive tract at various ages, as the data can be used as a reference for targeted intervention addressing intestinal *Bifidobacterium* composition as well as providing new ideas for probiotic products targeting various age groups.

## Figures and Tables

**Table 1:** Demographic characteristics of a cohorts.

Demographic Data	Age Group (y)			Values or No. (%)		
	0–17	18–65	66–108	0–17	18–65	66–108
Gender						
Male	96	80	33	96 (51%)	80 (39%)	33 (35%)
Female	69	118	61	69 (37%)	118 (58%)	61 (65%)
Not specified	23	6	0	23 (12%)	6 (3%)	0
Total no. of samples	188	204	94	–		
Total no. of strains	260	244	106	–		

**Table 2:** Phenotypic results for 6’-sialyllactose utilization in 21 strains of *Bifidobacterium*.

Strain	Age 0−17		Age 18−65		Age 66−108	
*B. bifidum*	FJSNJ1M3	++	FGSZY50M8	–	FHNFQ34M6	+
	FZJHZD4M4	+++	FHNFQ25M12	−	FHNFQ11M4	++
	FAHWH21M3	++	FGSYC45M3	−	FHNXY17M1	+
*B. longum* subsp. *infantis*	FGZ19I1M3	+++	FGZ6I2M6	+++	/	
	HeNJZ8M1	+++	FJ12WI1M14	+++	/	
	FGZ17I1M1	+++	FJ12WI1M1	++	/	
*B. breve*	JSWX17M1	++	/		FHNXY48M6	+++
	FJSZJ1M5	++	/		FCQNA20M1	++
	FBJCP1M6	+	/		FHNFQ34M6	++

Growth was classified as follows: −, negative (maximum OD600 < 0.2); +, low (OD600 from 0.2–0.5); ++, moderate (OD600 from 0.5–0.8); +++, high (OD600 > 0.8).

**Table 3:** Genome information for six *B. bifidum* strains used in this study.

Host	Bacterial Strain	Gene No.	Genome Size/Mb	G + C%	Level	Accession Number
Human feces (0–17)	*B. bifidum* FJSNJ1M3	1910	2.13	62.7	Scaffold	SRR13205608
	*B. bifidum* FZJHZD4M4	1959	2.17	62.6	Scaffold	SRR13205566
	*B. bifidum* FAHWH21M3	1899	2.12	62.7	Scaffold	SRR13205659
Human feces (18–65)	*B. bifidum* FGSZY50M8	1956	2.18	62.5	Scaffold	SRR13205646
	*B. bifidum* FHNFQ25M12	1985	2.12	62.6	Scaffold	SRR13205635
	*B. bifidum* FGSYC45M3	1932	2.13	62.7	Scaffold	SRR13205647

**Table 4:** Antibiotic resistance and susceptibility of six *B. bifidum* strains.

Strains	PEN	AMS	AMO	CLM	RIF	CEF	TET	TEC	VAN	OXA	CIP	GM	T/S	NEO	KAN	S	ATM	E
FJSNJ1M3	S	S	S	S	S	S	S	S	S	S	R	S	R	R	R	R	R	R
FZJHZD4M4	S	S	S	S	S	S	S	S	S	S	R	R	R	R	R	R	R	R
FAHWH21M3	S	S	S	S	S	S	S	S	S	S	R	R	R	R	R	R	R	R
FHNFQ25M12	S	S	S	S	S	S	S	S	S	S	R	R	R	R	R	R	R	R
FGSZY50M8	S	S	S	S	S	S	S	S	S	S	R	S	R	R	R	R	R	R
FGSYC45M3	S	S	S	S	S	S	S	S	S	S	R	R	R	R	R	R	R	R

Penicillin (PEN), Ampicillin sodium (AMS), Amoxicillin (AMO), Chloramphenicol (CLM), Rifampicin (RIF), Ceftizoxime (CEF), Tetracycline (TET), Teicoplanin (TEC), Vancomycin (VAN), Oxacillin (OXA), Ciprofloxacin (CIP), Gentamicin (GM), Trimethoprim (T/S), Neomycin (NEO), Kamamycin (KAN), Streptomycin (S), Aztreonam (ATM), Erythromycin (E). R = resistant, I = intermediate, S = susceptible.

## Data Availability

The data shown in this study are contained within the article.
